# Correction: Kinesin-3 mediated axonal delivery of presynaptic neurexin stabilizes dendritic spines and postsynaptic components

**DOI:** 10.1371/journal.pgen.1010291

**Published:** 2022-06-24

**Authors:** Devyn Oliver, Shankar Ramachandran, Alison Philbrook, Christopher M. Lambert, Ken C. Q. Nguyen, David H. Hall, Michael M. Francis

In the Funding section, the grant number from the funder NIH to author DHH is listed incorrectly. The correct grant number is: OD010943.
