# Supplementary figures and images for: Kinesin-3 mediated axonal delivery of presynaptic neurexin stabilizes dendritic spines and postsynaptic components
Source: PLoS Genet. 2022 Jan 28;18(1):e1010016. doi: 10.1371/journal.pgen.1010016 (PMC8827443; doi:10.1371/journal.pgen.1010016)

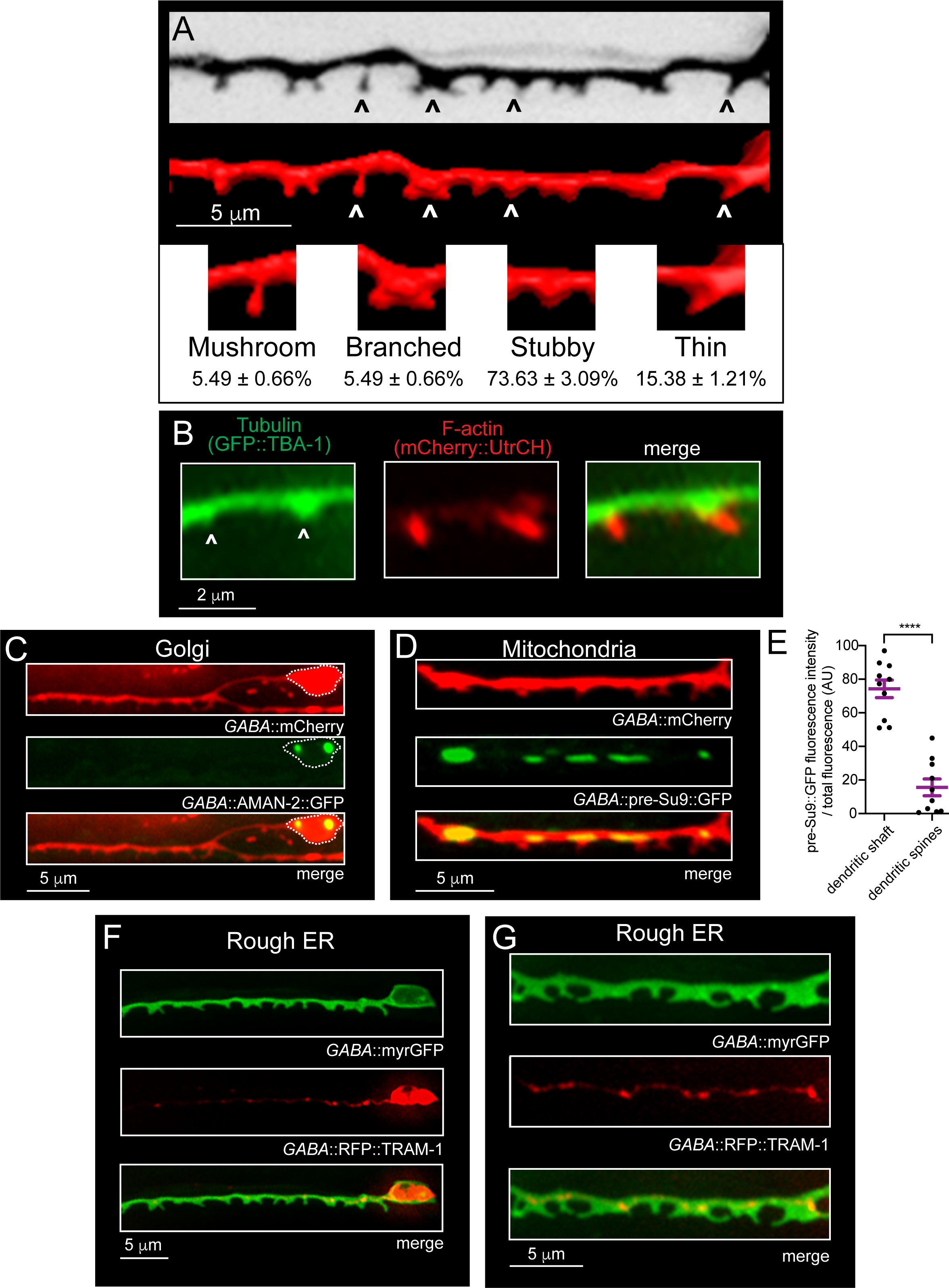

Supplement: S1 Fig — (A) Fluorescent image (top, inverted LUT) and 3D rendering (middle and insets) of DD dendritic spines from an animal expressing Pflp-13::mCherry, shows diverse spine morphologies. DD dendritic spines share morphological similarities with mammalian dendritic spines: mushroom (5.49 ± 0.66%), branched (5.49 ± 0.66%), stubby (73.63 ± 3.09%), and thin (15.38 ± 1.21%). Arrowheads, spine insets. n = 83 dendritic spines from 11 animals, measurements, percentage ± SD. (B) Fluorescent images of tubulin (Pflp-13::GFP::TBA-1) and F-actin (Pflp-13::mCherry::UtrCH) in DD neurons. White arrows indicate dendritic regions where tubulin contacts the base of a dendritic spine. (C) Fluorescent images of DD1 dendritic spines (Pflp-13::mCherry) and Golgi marker (Pflp-13::AMAN-2::GFP) in DD neurons. Golgi fluorescence is primarily restricted to the DD1 soma. Dotted white line traces the neuronal cell body. (D) Fluorescent images of DD1 dendritic spines (Pflp-13::mCherry) and mitochondria (Pflp-13::pre-Su9::GFP). Mitochondrial fluorescence is visible in the main dendritic processes of DD neurons near spines. (E) Quantification of mitochondria fluorescence intensity in dendritic shaft and dendritic spines. Bars, mean ± SEM. Student’s t-test, ****p<0.0001. (F) Fluorescent images of DD3 dendritic spines (Pflp-13::myrGFP) and rough endoplasmic reticulum (ER) (Pflp-13::RFP::TRAM). TRAM fluorescence is enriched in DD cell bodies but is also more weakly visible in the dendritic processes of DD neurons. (G) Fluorescent images of DD1 dendritic spines (Pflp-13::myrGFP) and rough endoplasmic reticulum (ER) (Pflp-13::RFP::TRAM). Fluorescence signal intensity was increased to better display process. (TIF) [file pgen.1010016.s002.tif]

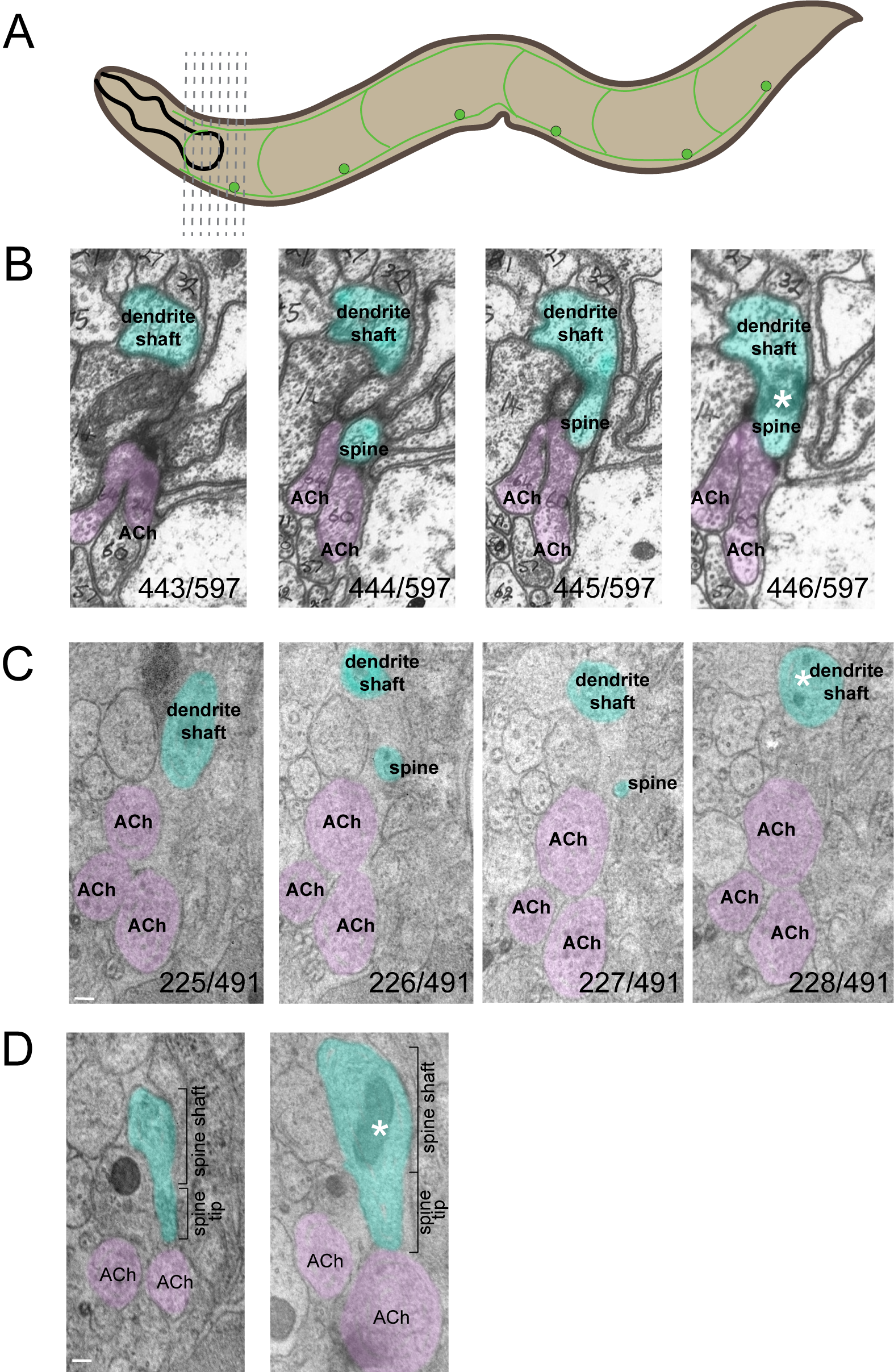

Supplement: S2 Fig — (A) Cartoon depiction of the anterior serial cross-sections used for electron microscopy studies of the ventral nerve cord, focusing on the dendrite of the DD1 neuron. Cross-sections in B are from White and colleagues [21] while sections in C and D are from this work. (B) Serial cross-sections (443-446/597) of the ventral nerve cord from N2U series [21]. Teal indicates DD1 dendrite and dendritic spines dipping into the ventral nerve cord to meet presynaptic cholinergic terminals (magenta). Asterisks indicates mitochondrion within the dendritic shaft, see S1 Fig. (C) Ventral nerve cord electron micrographs. Serial cross-sections (225-228/491) of the ventral nerve cord. Teal indicates DD1 dendrite and dendritic spines (226/491 and 227/491) dipping into the ventral nerve cord to meet presynaptic cholinergic terminals (presumably VA/VB neurons, magenta). Asterisks indicates mitochondria within the dendritic shaft, see S1 Fig. Scale bar, 100 nm. (D) Two representative micrographs from serial sections where the entire extent of the spine is visible are shown. Asterisk indicates mitochondrion within the dendritic shaft. Scale bar, 100 nm. (TIF) [file pgen.1010016.s003.tif]

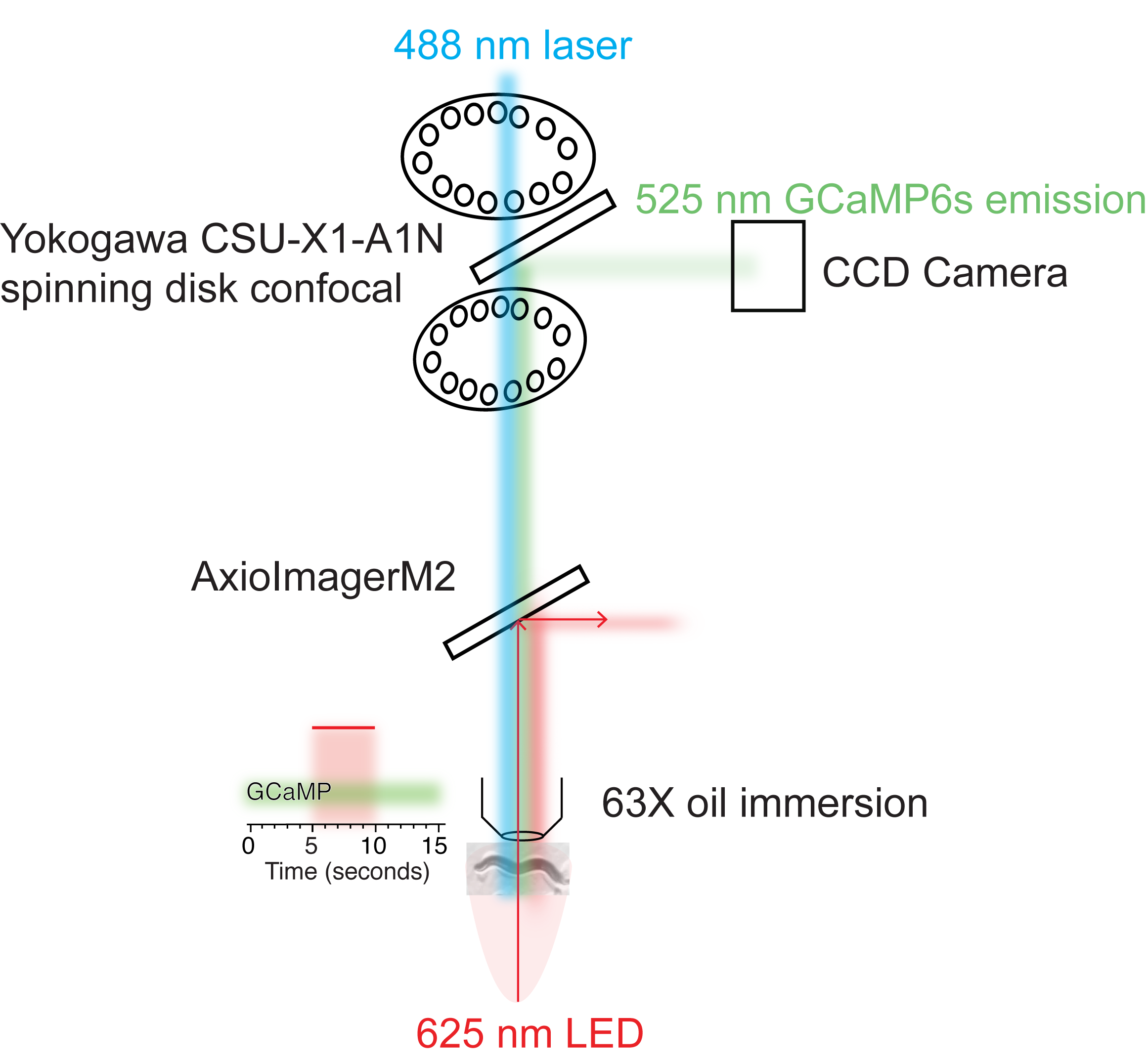

Supplement: S3 Fig — Imaging was performed using a Yokogawa CSU-X1-A1N spinning disk confocal system (Perkin Elmer) equipped with EM-CCD camera (Hamamatsu, C9100-50) and 63X oil immersion objective. Chrimson photoactivation (~30 mW/cm2) was achieved using a TTL-controlled 625 nm light guide coupled LED (Mightex Systems), permitting illumination of the entire immobilized animal, while simultaneously recording GCaMP6f fluorescence (excitation 488 nm, emission 525 nm). A 556 nm edge BrightLine single-edge short-pass dichroic beam splitter was positioned in the light path (Semrock) to prevent 625 nm light from reaching the camera. (TIF) [file pgen.1010016.s004.tif]

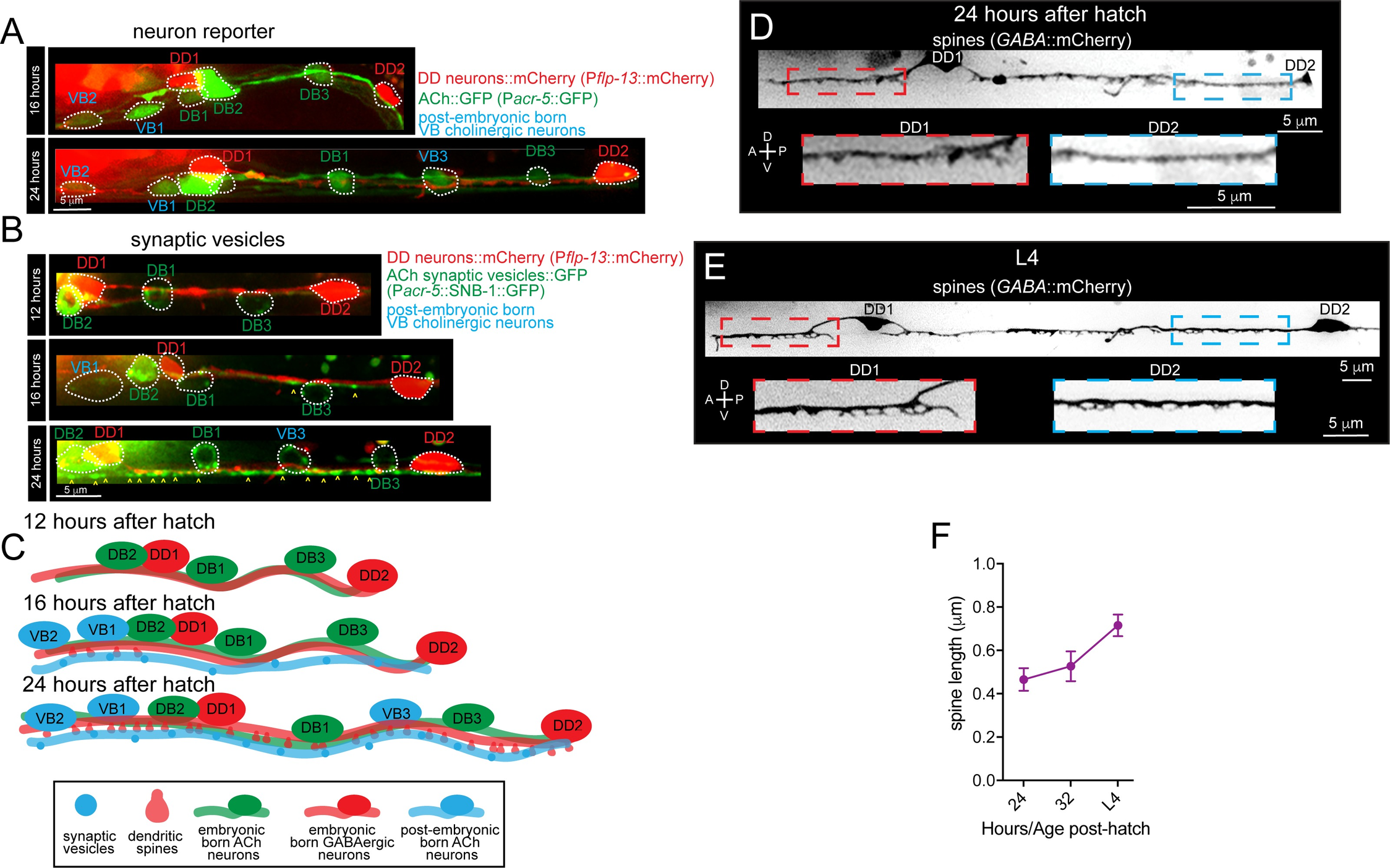

Supplement: S4 Fig — (A) Fluorescent images of B-type cholinergic neurons (DB/VB) (Pacr-5::GFP) (green) and DD GABAergic neurons (Pflp-13::mCherry) (red) at 16 and 24 hours after hatch. VB cholinergic neurons are born post-embryonically in an anterior to posterior order (blue). Note that at 16 hours after hatch Pacr-5::GFP fluorescence indicating VB1 and VB2 cell bodies is visible. By 24 hours after hatch Pacr-5::GFP fluorescence indicating VB3 is visible. White dotted circles outline the neuronal cell bodies. (B) Fluorescent images of the anterior ventral nerve cord in animals co-expressing the synaptic vesicle marker Pacr-5::SNB-1::GFP in B-type cholinergic neurons (DB/VB) (green) with Pflp-13::mCherry labeling DD GABAergic neurons (red) at 12, 16, and 24 hours after hatch. At 12 hours, little SNB-1::GFP fluorescence is visible in the ventral nerve cord. Embryonic born, dorsally directed B-type (DB) cholinergic motor neurons predominantly make synaptic contacts in the dorsal nerve cord. Ventrally-directed, B-type (VB) cholinergic motor neurons are born post-embryonically and have not yet completed their maturation at this time. SNB-1::GFP fluorescence in the ventral nerve cord is faintly visible by 16 hrs after hatch and becomes more prominent by 24 hrs after hatch, coincident with maturation of VB motor neurons. White dotted circles indicate outlines of neuronal cell bodies. Yellow arrows indicated presynaptic vesicle clusters, SNB-1. (C) Cartoon representation of the developmental timing of post-embryonic born ventral cholinergic motor neurons, spine outgrowth, and cholinergic synaptic vesicle localization. (D-E) Fluorescent images (inverted LUT) of DD1 and DD2 dendritic spines 24 hours after hatch (D) and at L4 (~42–50 hours after hatch) stage (E). Animals express Pflp-13::mCherry to label dendritic spines. Red dashed rectangle indicates inset anterior to DD1 soma. Blue dashed rectangle indicates inset anterior to DD2 soma. Note that DD1 spines form prior to DD2 spines. (F) Quantif [file pgen.1010016.s005.tif]

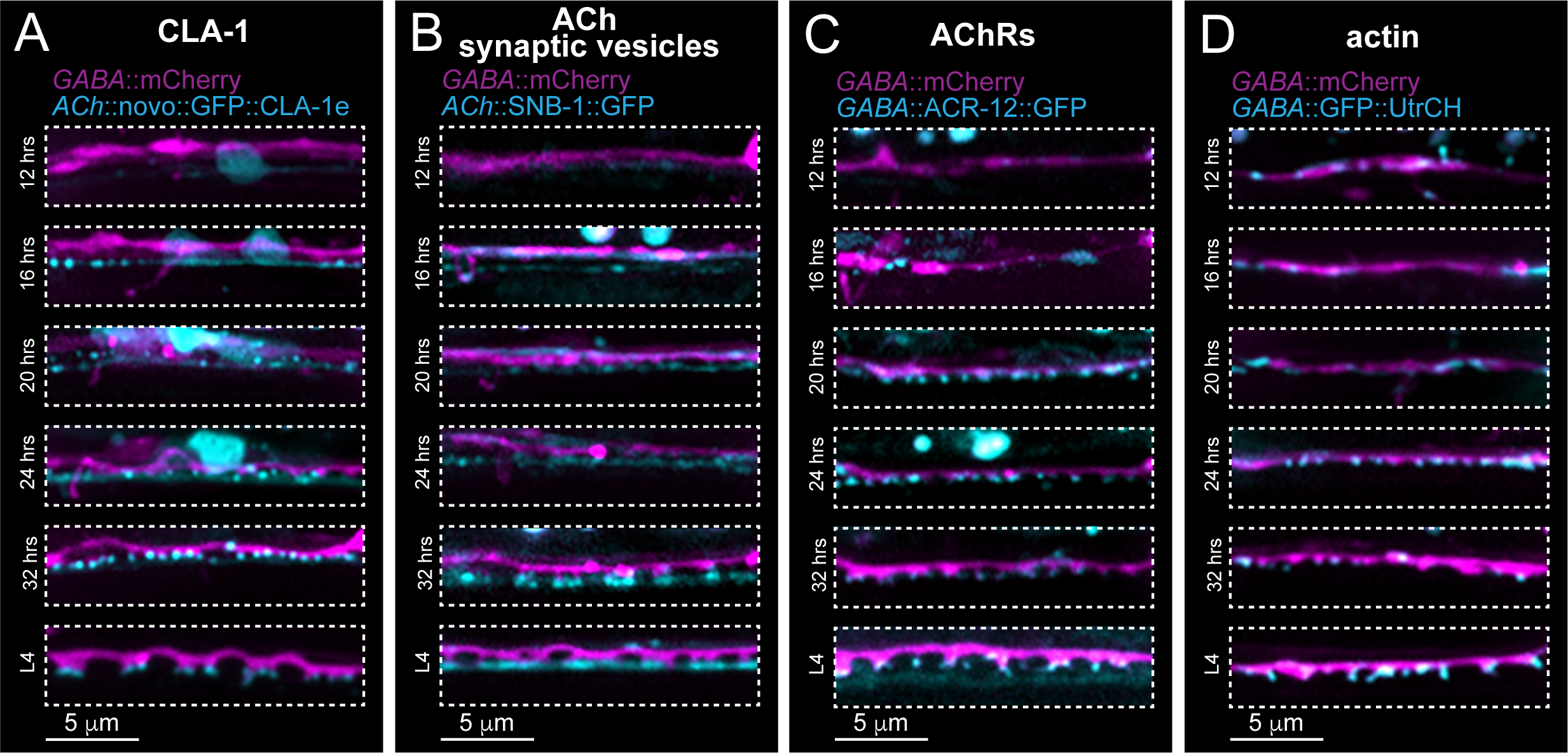

Supplement: S5 Fig — Fluorescent images of GABAergic dendrites at 12, 16, 20, 24, and 32 hours after hatch and at L4 stage (~42–50 hours post-hatch). Animals express a GABAergic dendrite marker (Pflp-13::mCherry) with either (A) presynaptic active zone marker (Punc-17β::GFPnovo2::CLA-1e), (B) synaptic vesicle marker (Pacr-5::SNB-1::GFP) or postsynaptic receptors (C) (Punc-47::ACR-12::GFP) or (D) F-actin (Pflp-13::GFP::UtrCH) (Punc-17β::GFPnovo2::CLA-1e). Images are pseudo colored to indicate spines (magenta) and synaptic components (CLA-1, vesicle clusters, F-actin, or AChRs) (cyan). (TIF) [file pgen.1010016.s006.tif]

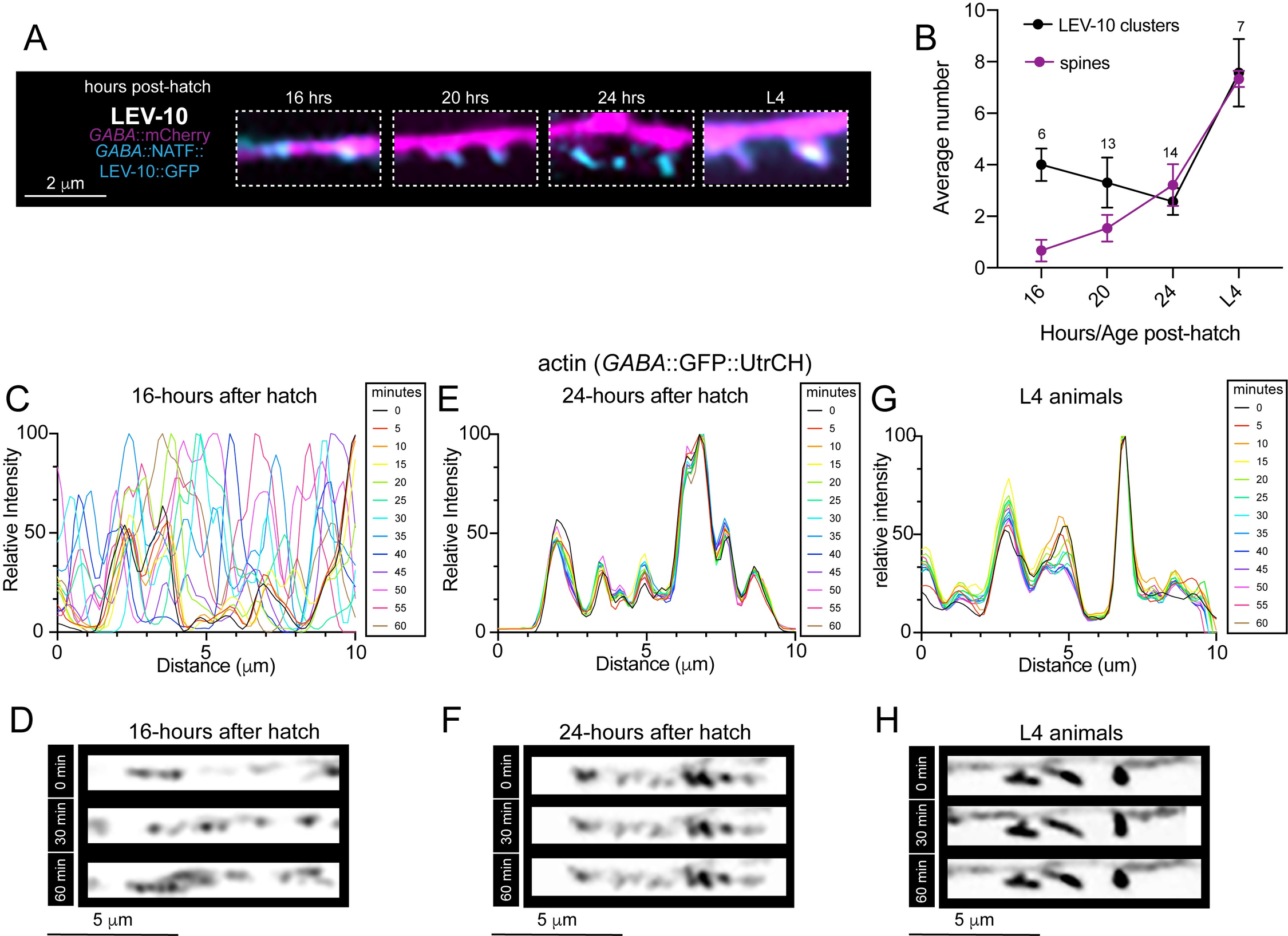

Supplement: S6 Fig — (A) Fluorescent confocal images showing dendritic spines (magenta) and the postsynaptic CUB transmembrane domain LEV-10 (cyan). Animals express Pflp-13::mCherry with DD neuron specific LEV-10::GFP11x7 using a strategy for cell-specific labeling of endogenous LEV-10 (NATF) [39]. (B) Quantification of the number of DD dendritic spines and LEV-10 clusters at 16, 20, and 24 hours after hatch, and L4 stage (~42–50 hours post-hatch). (C) (C, E, G) Line scans displaying relative fluorescence intensity of F-actin (Pflp-13::GFP::UtrCH) at 16 hours after hatch (C), 24 hours after hatch (E), and L4 (~42–50 hours after hatch) animals (G). Each color indicates a line scan of fluorescence intensity for the same DD dendrite ROI acquired at 5-minute intervals. Note the variable distribution of fluorescence intensities across line scans from images acquired near 16 hours after hatch compared to later time points, indicating increased F-actin dynamics during early development. (D, F, H) Confocal images (inverted LUT) showing Pflp-13::GFP::UtrCH fluorescence (labeling F-actin) in the DD dendrite of 16 (D), or 24 (F) hours after hatch, or at L4 stage (~42–50 hours after hatch) (H). For each, sequences of fluorescent images separated by 30 minutes are shown. (TIF) [file pgen.1010016.s007.tif]

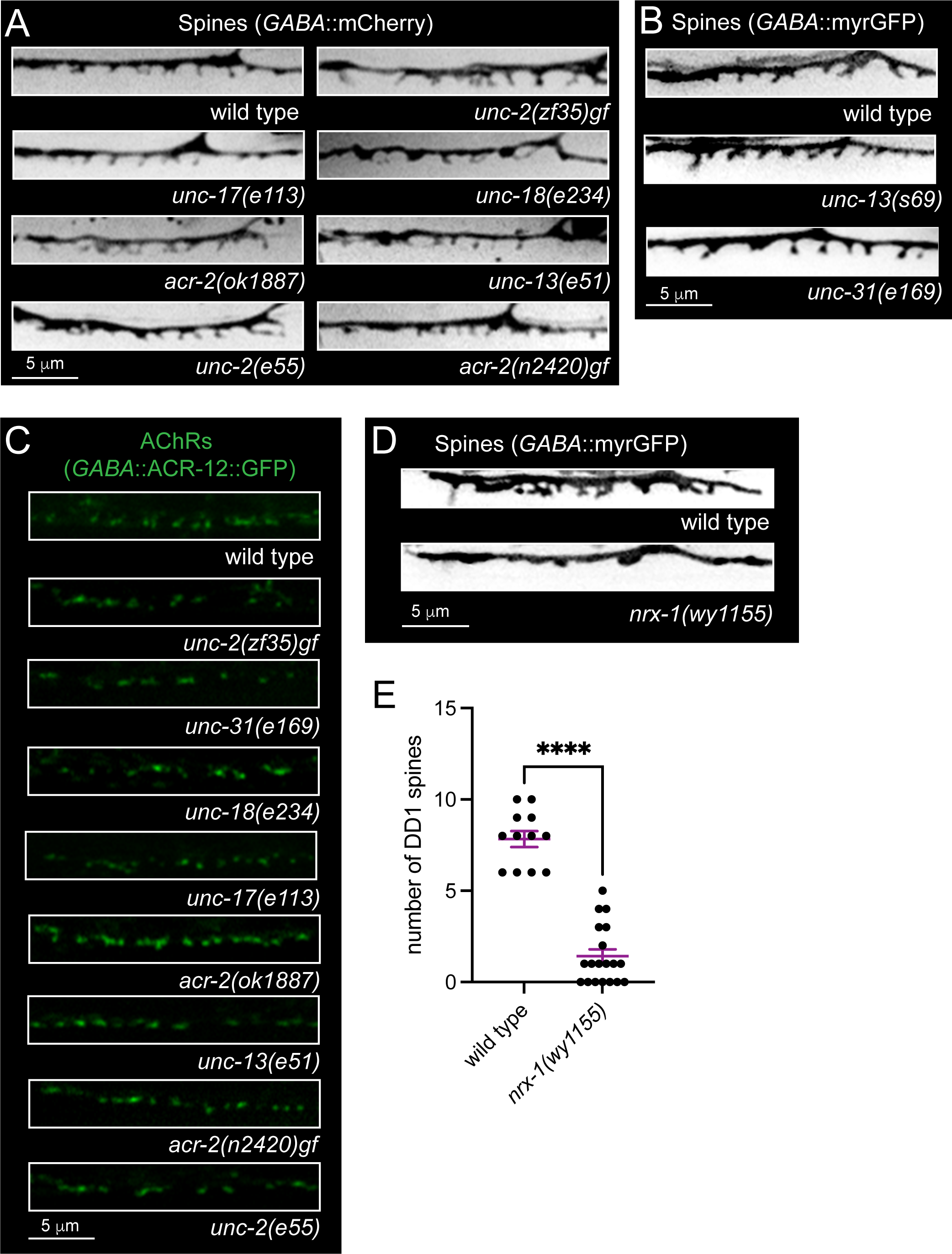

Supplement: S7 Fig — (A, B) Fluorescent confocal images (inverted LUT) of DD1 dendritic spines (Pflp-13::mCherry (A) or Pflp-13::myrGFP (B)) in L4 stage wild type and selected mutant strains where synaptic activity is affected. (A) Fluorescent confocal images of AChR clusters (Pflp-13::ACR-12::GFP) in the DD dendrite of L4 stage wild type and selected mutant strains where synaptic activity is affected. (B) Fluorescent confocal images (inverted LUT) of DD1 dendritic spines (Pflp-13::myrGFP) in L4 stage wild type and nrx-1(wy1155) null mutants. (C) Quantification of the number of DD1 dendritic spines in L4 wild type and nrx-1(wy1155) null mutants. Student’s t-test, ****p<0.0001. Bars, mean ± SEM. (TIF) [file pgen.1010016.s008.tif]

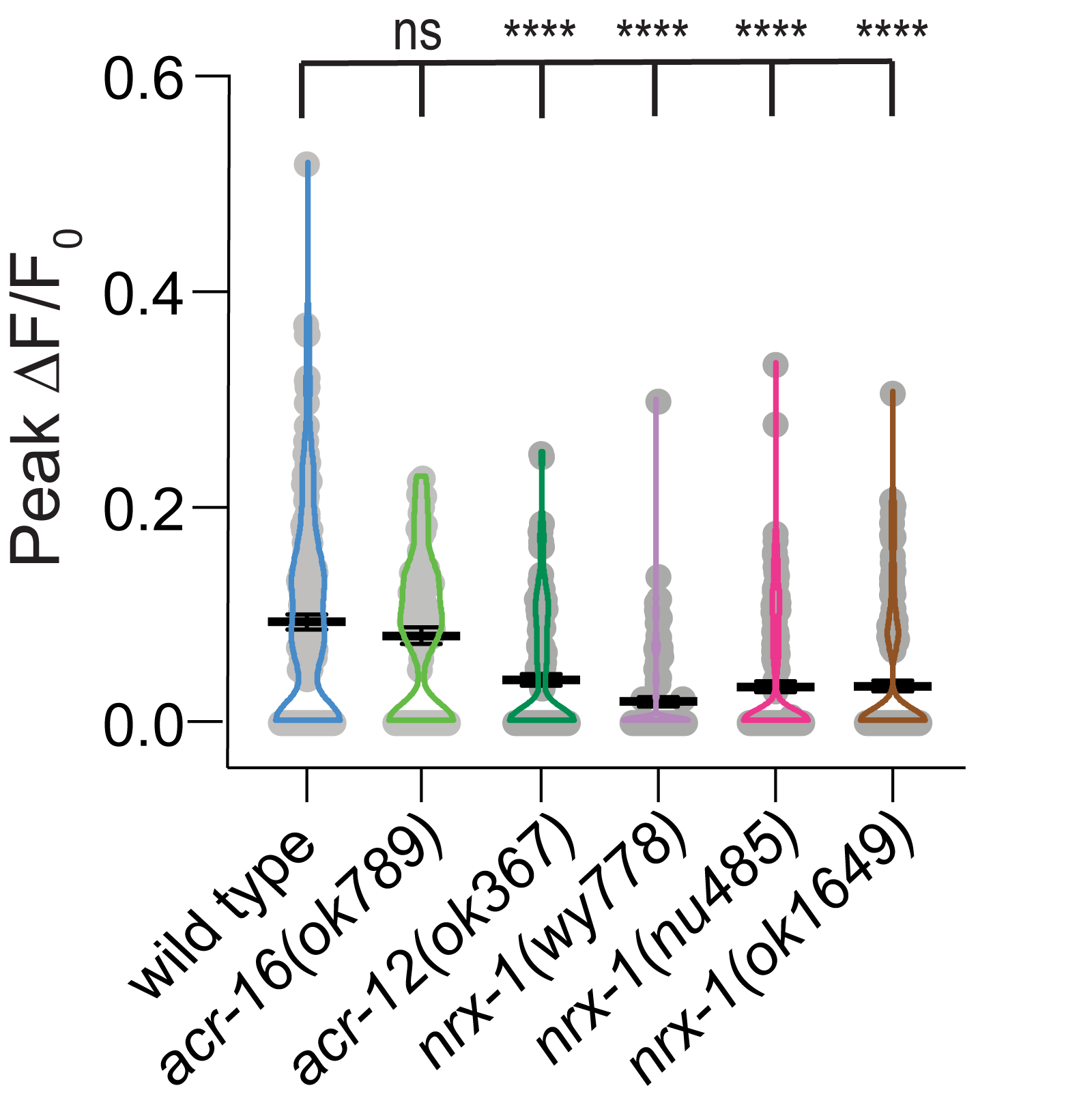

Supplement: S8 Fig — Scatter plot showing peak ΔF/Fo responses measured from GABAergic DD motor neuron dendrites during a 5s period of cholinergic photostimulation in wild type, acr-16(ok789); acr-12(ok367), nrx-1(wy778), nrx-1(nu485), and nrx-1(ok1649) mutants. All genotypes co-express Pflp-13::myrGCaMP6f::SL2::mCherry for measurement of dendritic calcium responses with Pacr-2::Chrimson for cholinergic neuron depolarization. Dendritic calcium responses were not significantly affected by deletion of the homomeric nAChR subunit acr-16, but were significantly reduced by mutation of either acr-12 or nrx-1. Bars indicate mean ± SEM. One-way ANOVA, Dunnett’s multiple comparisons, ****p<0.0001. Wild type control is the same as Fig 1H. n ≥ 10 animals. (TIF) [file pgen.1010016.s009.tif]

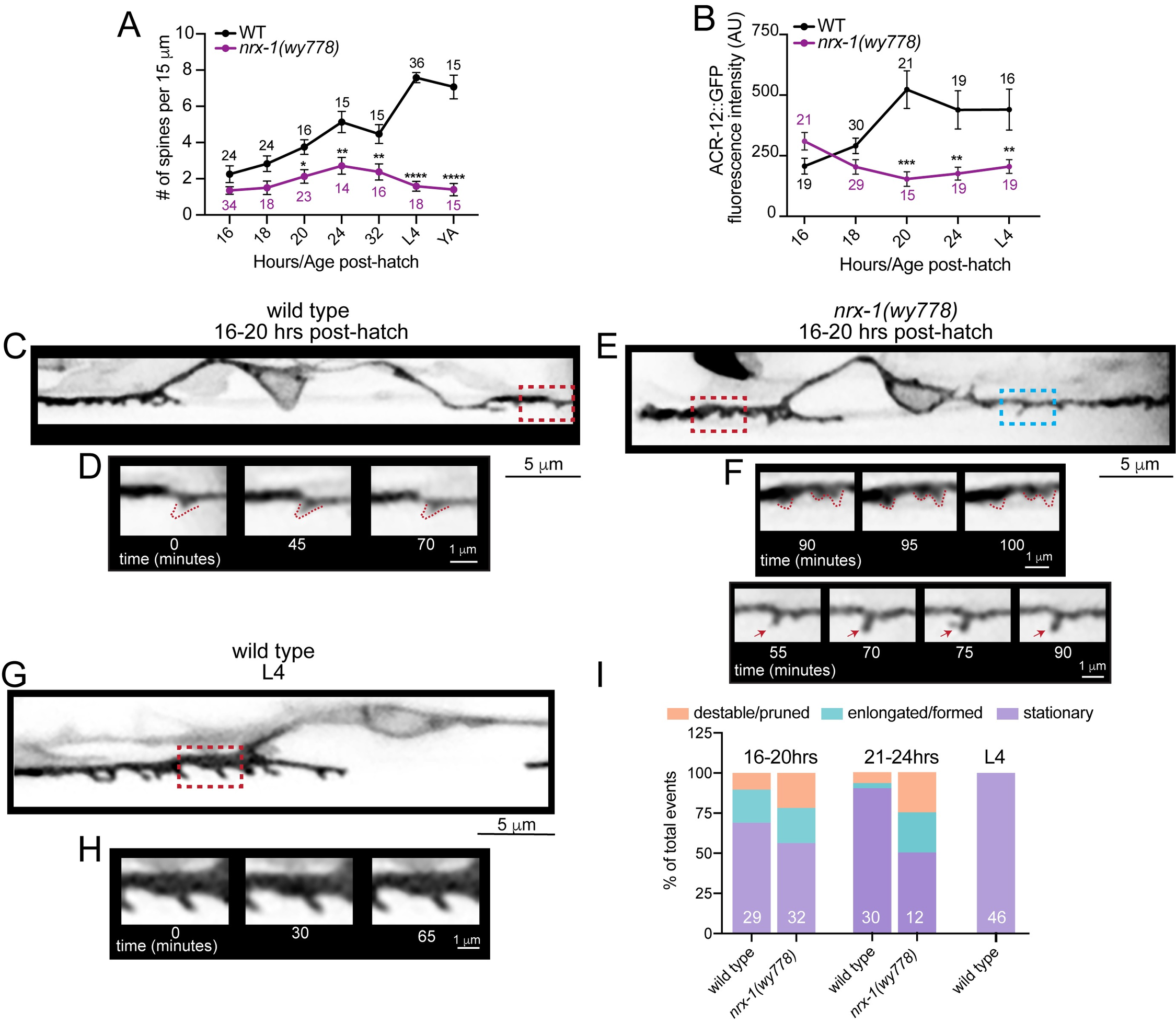

Supplement: S9 Fig — (A) Quantification of the number of DD spines/15 μm in wild type (black) and nrx-1(wy778) (purple) animals 16, 20, 24, 32 hours after hatch and at L4 (~42–50 hours after hatch) and young adult (YA) (~52–56 hours after hatch) stages. Two-way ANOVA, Sidak’s multiple comparisons test, * p<0.05, **p<0.01, ****p<0.0001. Data points indicate mean ± SEM. Numbers indicate animals quantified for each timepoint. (B) Quantification of ACR-12::GFP fluorescence intensity from DD dendrites of wild type (black) and nrx-1(wy778) (purple) animals at 16, 20, and 24 hours after hatch and L4 stage (~42–50 hours post-hatch). Two-way ANOVA, Sidak’s multiple comparisons test, **p<0.01, ***p<0.001. Data points indicate mean ± SEM. (C) Fluorescent image (inverted LUT) of DD dendritic spines at timepoint zero in a wild type animal 16–20 hours after hatch. Dashed red box indicates region shown in S9D Fig. (D) Fluorescent images (inverted LUT) of an individual wild type DD dendritic spine (Pflp-13::mCherry) at 0, 45, and 70 minute timepoints during live imaging of area indicated by red box in S9C Fig. Red dashed line indicates largest extent of spine outgrowth. (E) Fluorescent image (inverted LUT) of nrx-1(wy778) mutant DD1 dendritic spines (Pflp-13::mCherry) at timepoint zero acquired 16–20 hours after hatch. Dashed red and blue boxes indicate regions shown in S9F Fig. (F) Fluorescent images (inverted LUT) of individual nrx-1(wy778) mutant DD dendritic spines (Pflp-13::mCherry) at (top) 90. 95, and 100 minute timepoints during live imaging of area indicated by red box in S9E Fig and (bottom) at 555, 70, 75, 90 minute timepoints during live imaging of area indicated by blue box in S9E Fig. Red dashed line indicates largest extent of spine outgrowth. Red arrows indicate spine dynamics. (G) Fluorescent image (inverted LUT) of wild type DD dendritic spines (Pflp-13::mCherry) at timepoint zero at L4 stage. Dashed red box indicates region shown in S9H Fig. (H) Fluorescent images (inverted LUT) of a [file pgen.1010016.s010.tif]

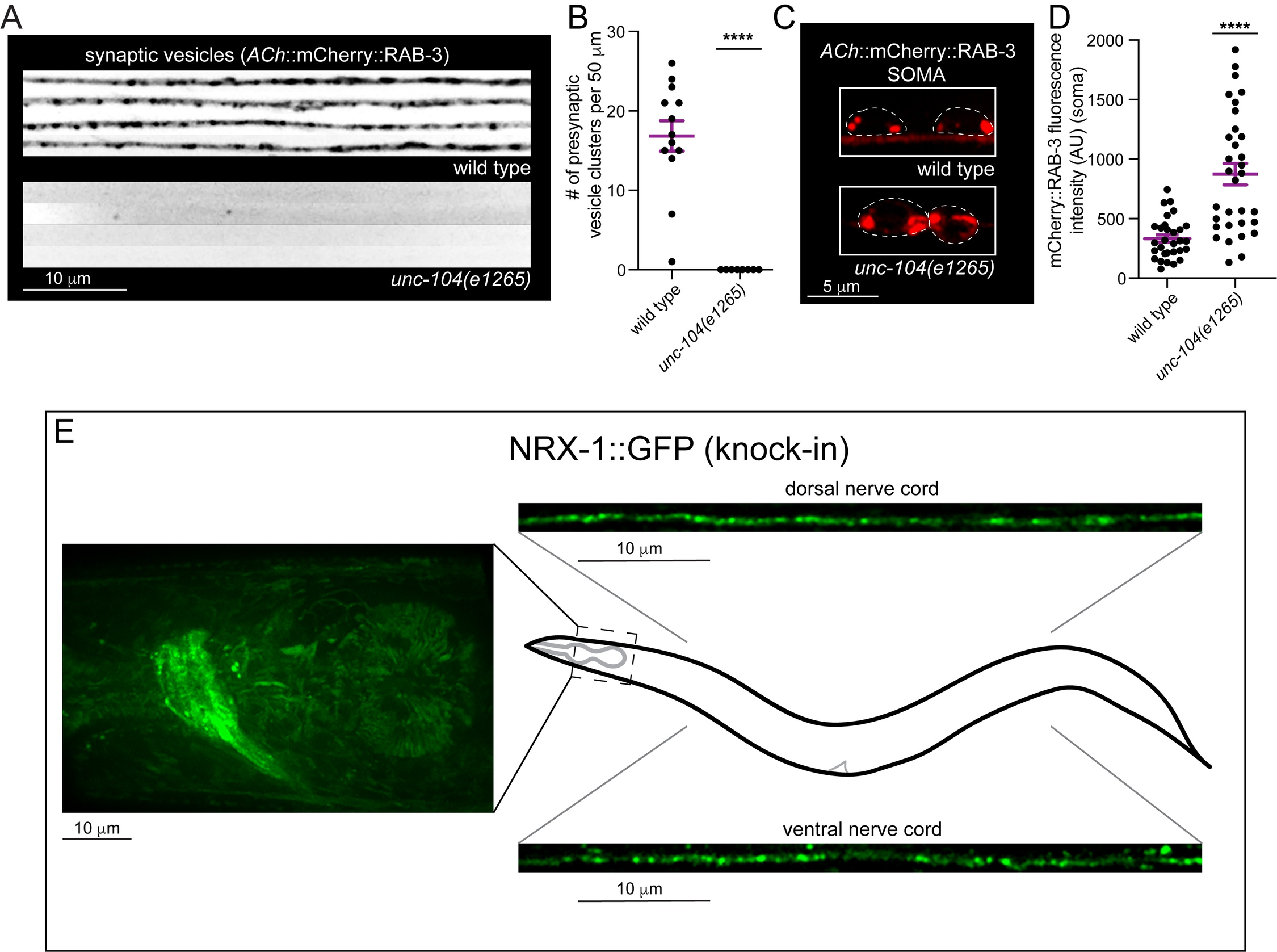

Supplement: S10 Fig — (A) Fluorescent images of cholinergic synaptic vesicles (Pacr-2::mCherry::RAB-3) in the dorsal nerve cord of young adult wild type and unc-104(e1265) mutants. Images on each line are from different animals (4 are shown for each genotype). (B) Quantification of cholinergic synaptic vesicles (Pacr-2::mCherry::RAB-3) per 50 μm of the dorsal nerve cords of wild type and unc-104(e1265) mutants. Student’s t-test, ****p<0.0001. Bars, mean ± SEM. (C) Fluorescent images of the soma in wild type and unc-104(e1265) mutants expressing cholinergic vesicle reporter (Pacr-2::mCherry::RAB-3). Dotted white lines outline the cell body. (D) Quantification of soma cholinergic synaptic vesicle fluorescence intensity (AU) (Pacr-2::mCherry::RAB-3) of wild type and unc-104(e1265) mutants. Student’s t-test, ****p<0.0001. Bars, mean ± SEM. (E) NRX-1::GFP localizes within neuronal processes of the nerve ring and shows punctate localization in processes of the ventral and dorsal nerve cords of L4 stage worms. (TIF) [file pgen.1010016.s011.tif]

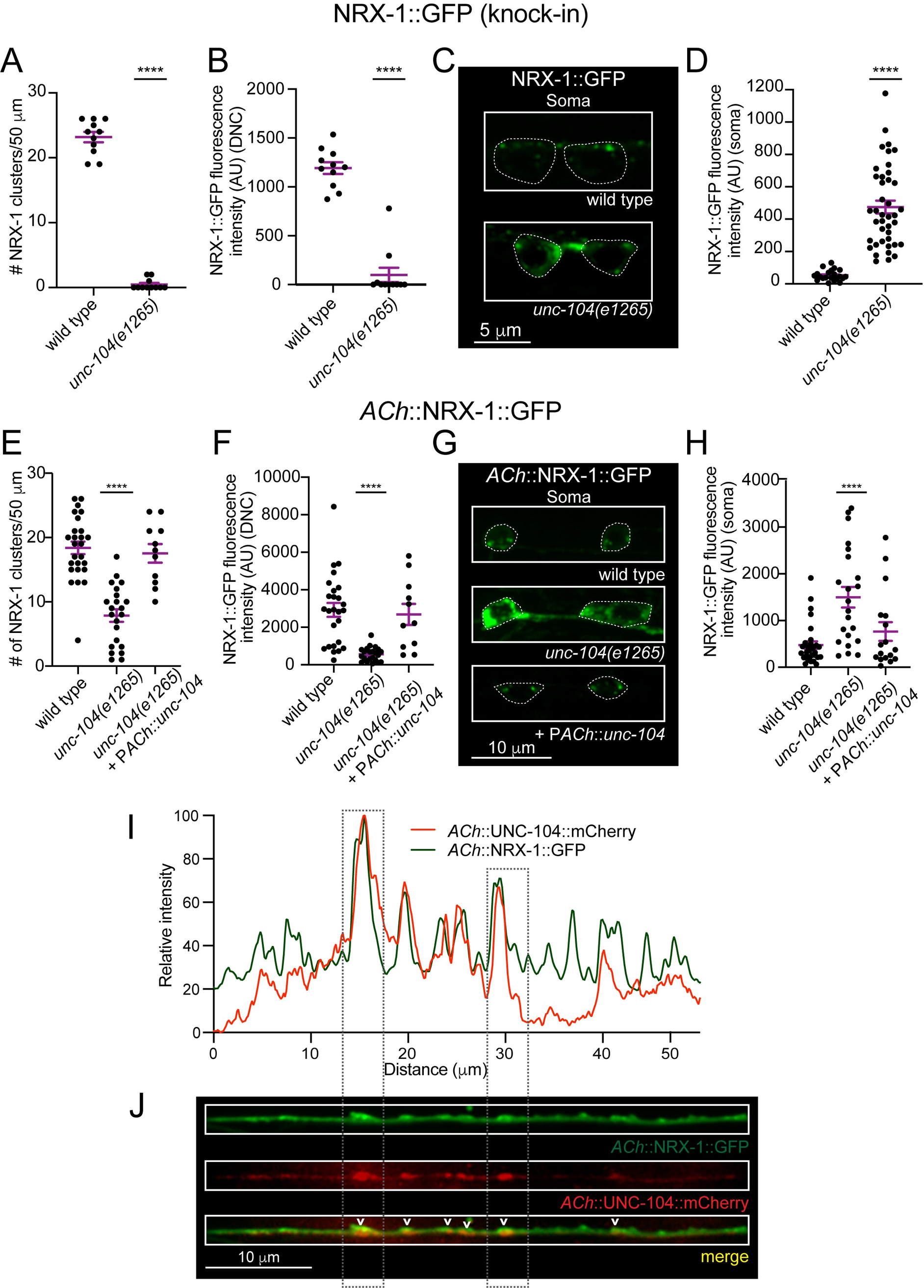

Supplement: S11 Fig — (A) Quantification of NRX-1::GFP (endogenous knock-in) clusters in a 50 μm region of the dorsal nerve cord in wild type and unc-104(e1265) animals. Bars, mean ± SEM. Student’s t-test, ****p<0.0001. n ≥ 11 animals. (B) Quantification of NRX-1::GFP (endogenous knock-in) axon fluorescence intensity in a 50 μm region of the dorsal nerve cord. Bars, mean ± SEM. Student’s t-test, ****p<0.0001. n ≥ 11 animals. (C) Fluorescent images of NRX-1::GFP (endogenous knock-in) in somas of wild type and unc-104(e1265) mutants. Dotted white lines outline the neuronal cell body. (D) Quantification of NRX-1::GFP soma fluorescence intensity. Bars, mean ± SEM. Student’s test, ****p<0.001. (E) Quantification of the number of NRX-1::GFP (Punc129::NRX-1::GFP) clusters in a 50 μm region of the dorsal nerve cord in wild type, unc-104(e1265), and unc-104(e1265) mutants rescued with cholinergic expression of wild type unc-104. Bars, mean ± SEM. One-way ANOVA, Dunnett’s multiple comparisons test, ****p<0.0001. n ≥ 11 animals. (F) Quantification of NRX-1::GFP fluorescence intensity in cholinergic axons (Punc129::NRX-1::GFP) of a 50 μm region of the dorsal nerve cord. Bars, mean ± SEM. One-way ANOVA, Dunnett’s multiple comparisons test, ****p<0.0001. n ≥ 11 animals. (G) Fluorescent images of NRX-1::GFP in cholinergic somas of wild type, unc-104(e1265) and unc-104(e1265) mutants rescued with cholinergic expression of wild type unc-104. Dotted white lines outline the neuronal cell body. (H) Quantification of NRX-1::GFP fluorescence intensity in cholinergic somas. Bars, mean ± SEM. One-way ANOVA, Dunnett’s multiple comparisons test, ****p<0.001. (I) Line scans depicting relative fluorescent intensity of NRX-1::GFP (green) and UNC-104::mCherry (red) for a 50 μm region of the dorsal nerve cord. Gray dotted rectangles indicate corresponding puncta in S11J Fig. (J) Fluorescent images of the dorsal nerve cord in an adult animal expressing NRX-1::GFP (Punc-129::NRX-1::GFP) and UNC-104::mCherry (Punc-129::U [file pgen.1010016.s012.tif]

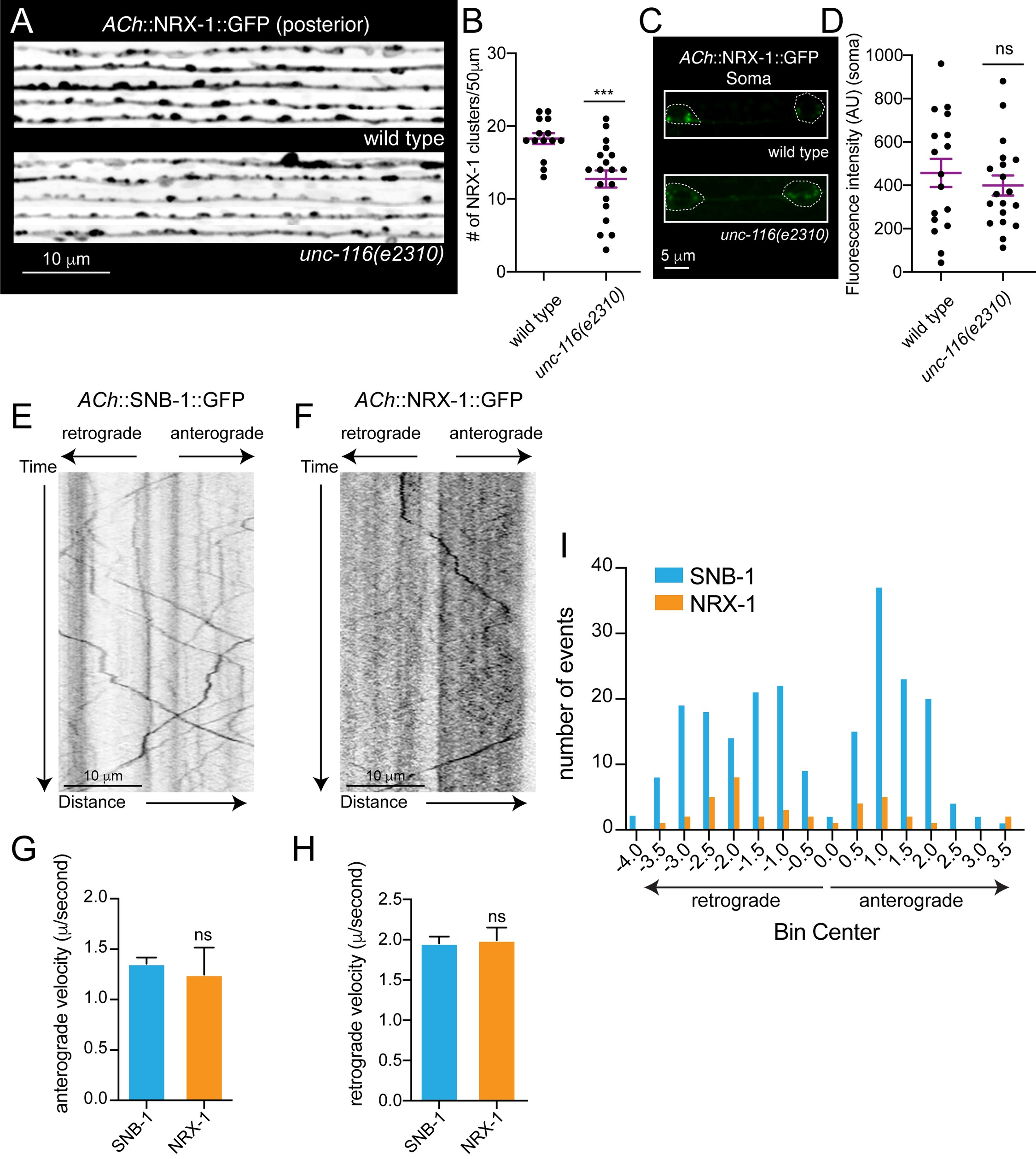

Supplement: S12 Fig — (A) Fluorescent images (inverted LUT) of NRX-1::GFP (Punc-129::NRX-1::GFP) from the dorsal nerve cord of wild type and unc-116(e2310) mutants. Images on each line are from different animals (5 are shown for each genotype). (B) Quantification of the number of NRX-1 clusters (Punc-129::NRX-1::GFP) in a 50 μm region of the dorsal nerve cord of wild type and unc-116(e2310) animals. Bars, mean ± SEM. Student’s t-test, ***p<0.001. n ≥ 14 animals. (C) Fluorescent images of NRX-1 (Punc-129::NRX-1::GFP) from cholinergic neuron somas of wild type and unc-116(e2310) mutants. Dotted white lines outline cell bodies. (D) Quantification of fluorescence intensity of NRX-1 (Punc-129::NRX-1::GFP) from cholinergic somas of wild type and unc-116(e2310) mutants. Bars, mean ± SEM. Student’s t-test, ns, not significant. (E, F) Kymographs of synaptic vesicle (Punc-129::SNB-1::GFP) (E) and NRX-1 (Punc-129::NRX-1::GFP) (F) trafficking events recorded from cholinergic neuron commissures. (G) Quantification of the anterograde velocity (μm/second) of SNB-1::GFP (blue) and NRX-1::GFP (orange) along the axonal commissure. ns, not significant. n ≥ 10 animals for all panels. (H) Quantification of the retrograde velocity (μm/second) of SNB-1::GFP (blue) and NRX-1::GFP (orange) along the axonal commissure. ns, not significant. (I) Quantification of the total number of SNB-1::GFP (blue) and NRX-1::GFP (orange) trafficking events binned into retrograde and anterograde directions. Note that NRX-1 trafficking events occur significantly less frequently than SNB-1 events. (TIF) [file pgen.1010016.s013.tif]

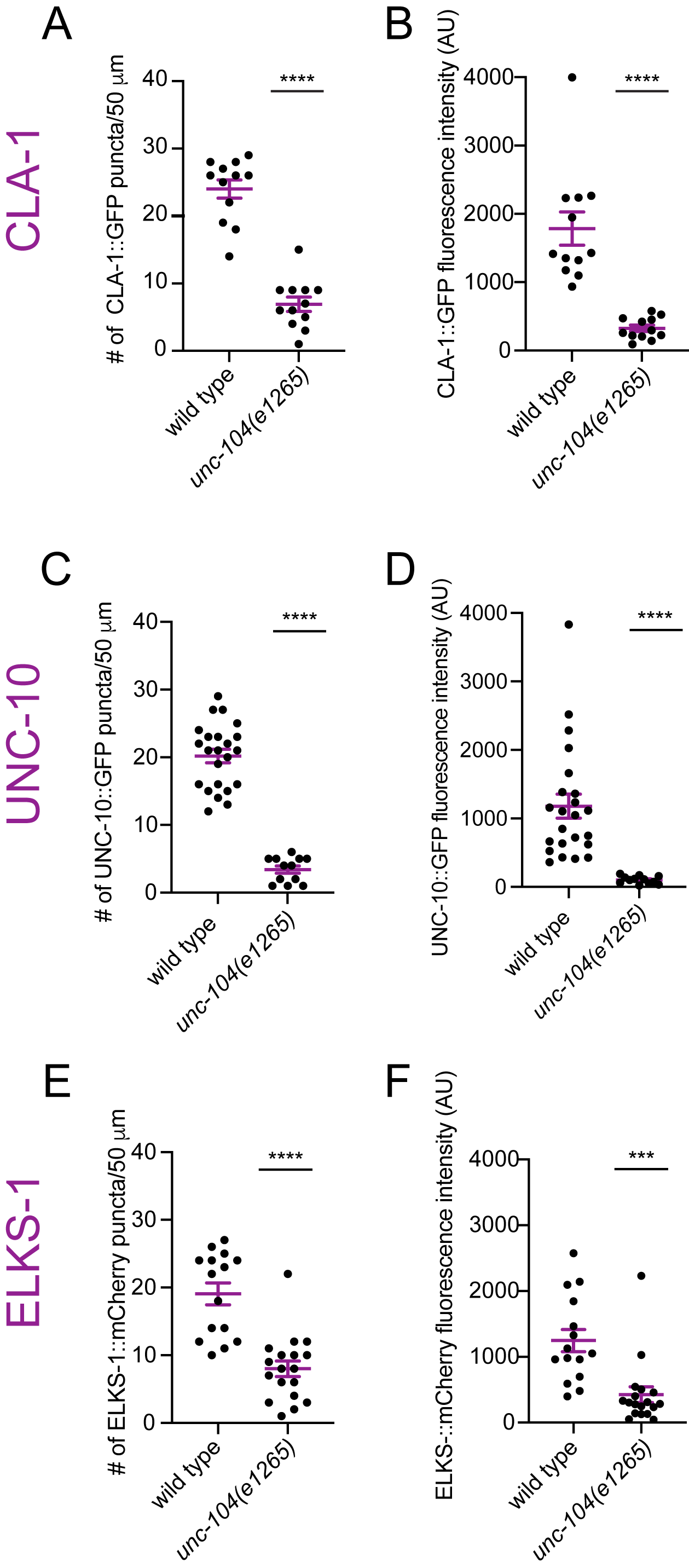

Supplement: S13 Fig — (A) Scatterplot of CLA-1::GFP puncta number in a 50 μm region of the dorsal nerve cord in wild type and unc-104(e1265) animals. Student’s t-test, ****p<0.0001. Bars, mean ± SEM. These data correspond to quantification in Fig 4C. (B) Scatterplot of CLA-1::GFP fluorescence intensity in a 50 μm region of the dorsal nerve cord in wild type and unc-104(e1265) animals. Student’s t-test, ****p<0.0001. Bars, mean ± SEM. (C) Scatterplot of UNC-10::GFP puncta number in a 50 μm region of the dorsal nerve cord in wild type and unc-104(e1265) animals. Student’s t-test, ****p<0.0001. Bars, mean ± SEM. These data correspond to quantification in Fig 4C. (D) Scatterplot of UNC-10::GFP fluorescence intensity in a 50 μm region of the dorsal nerve cord in wild type and unc-104(e1265) mutants. Student’s t-test, ****p<0.0001. Bars, mean ± SEM. (E) Scatterplot of ELKS-1::mCherry puncta number per 50 μm of the dorsal nerve cord in wild type and unc-104(e1265) animals. Student’s t-test, ****p<0.0001. Bars, mean ± SEM. These data correspond to quantification in Fig 4C. (F) Scatterplot of ELKS-1::mCherry fluorescence intensity in a 50 μm region of the dorsal nerve cords of wild type and unc-104(e1265) mutants. Student’s t-test, ***p<0.001. Bars, mean ± SEM. (TIF) [file pgen.1010016.s014.tif]

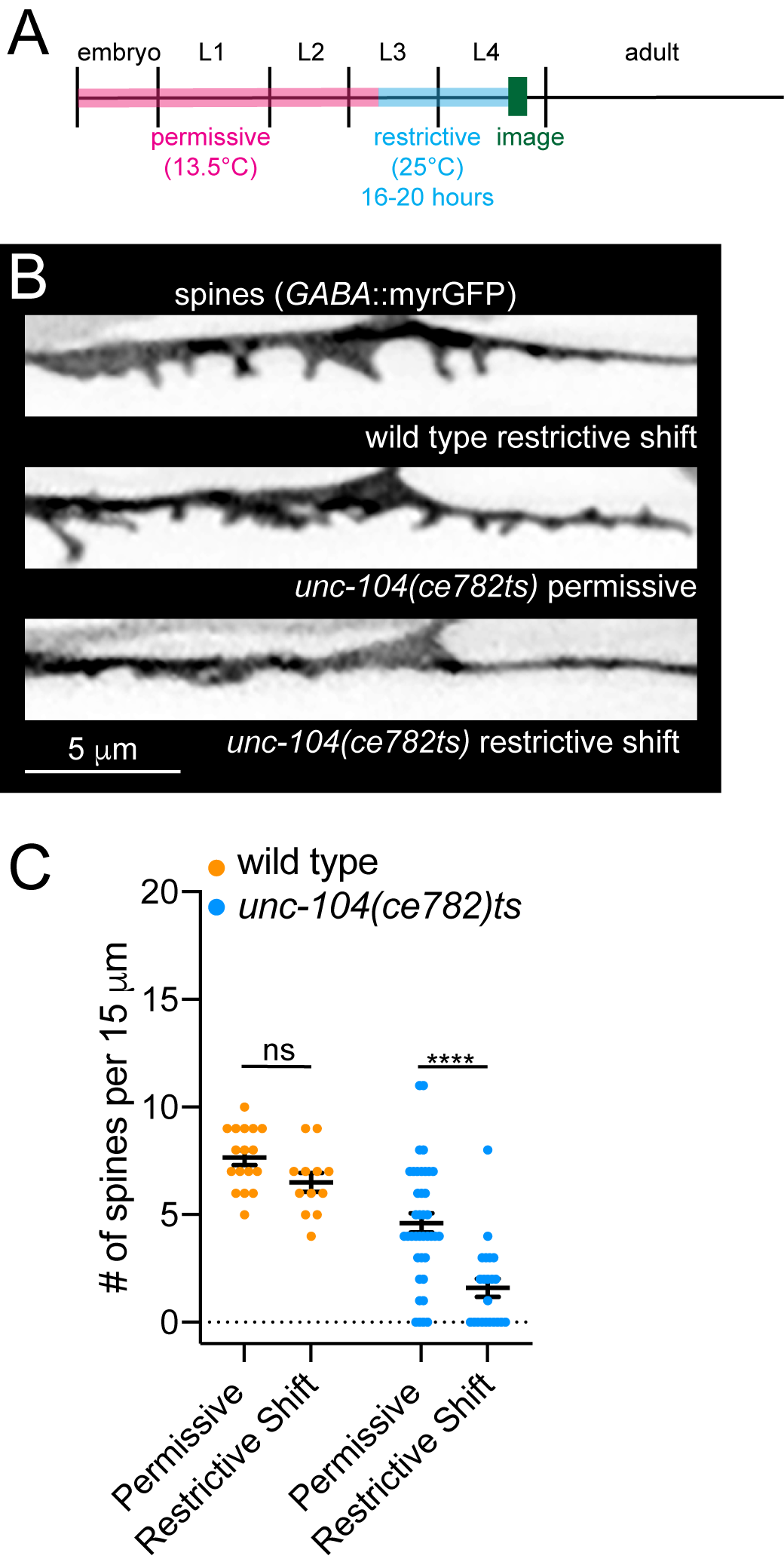

Supplement: S14 Fig — (A) Cartoon depiction of experimental timeline. Animals were grown at 13.5°C until L3 stage (~approx. 120 hours in unc-104(ce782) mutants, 96 hours in wild type animals) before shifting animals to their restrictive temperature of 25°C for 16–20 hours and imaging. (B) Fluorescent images of DD spines (Pflp-13::myrGFP) from wild type (top) or unc-104(ce782)ts animals grown continuously at the permissive temperature (13.5°C) (middle panel) or shifted to the restrictive temperature (25°C) for 16–20 hours before imaging. (C) Quantification of DD spines per 15 μm from wild type and unc-104(ce782)ts mutants. Two-way ANOVA, Tukey’s multiple comparisons test, ****p<0.0001, n ≥ 12 animals. Data points indicate mean ± SEM. (TIF) [file pgen.1010016.s015.tif]
