# Supplementary material for: Kinesin-3 mediated axonal delivery of presynaptic neurexin stabilizes dendritic spines and postsynaptic components
Source: PLoS Genet. 2022 Jan 28;18(1):e1010016. doi: 10.1371/journal.pgen.1010016 (PMC8827443; doi:10.1371/journal.pgen.1010016)
Supplement: Attachment — Submitted filename: Response to Reviewers FINAL_Oliver.pdf [file pgen.1010016.s024.pdf]

Response to Reviewers

We thank the reviewers for their helpful comments and suggestions. We address each of their specific points below. We hope our responses and revisions will clarify the issues raised.

Reviewer #1: This manuscript examines the developmental timeline and molecular mechanisms that control the formation and stabilization of Spine Like Portrusions (SLPs) in C.elegans. Previous work from the Francis lab identified SPLs and provided their initial characterization. The current manuscript follows up on that work. The authors describe the timeline of spine formation, looking at cytoskeletal components and specific organelles. They then go on to focus on nrx-1 (which they have previously shown plays a role in SLP formation. They provide data that Neurexin's main role is in synapse stabilization, rather than early formation. They also describe Neurexin as an UNC-104/kinesin-3 cargo and suggest that it is actually the main UNC-104 cargo that is important (on the presynaptic side) for SLP maintenance.

Overall, I strongly support publication. The manuscript provides a nice addition to our knowledge of SLP development in *C.elegans*. While the results of many of the experiments seem more confirmatory than novel, the scope and depth of the work certainly warrant publication in my opinion. In addition, the manuscript is clearly written and overall attention to details is high. There are several issues which should be addressed:

We thank the reviewer for their positive assessment.

1. The tubulin marker looks quite diffuse and possibly not integrated into MTs. This is likely because of the C-ter tag. TBA-1 should be tagged on the N terminus. My personal view is that a partial analysis of microtubules (i.e. not studying their dynamics, polarity and organization), even with a better marker, does not contribute significantly to the paper and I would suggest removing it. Otherwise, to argue that the SLPs are similar to spines in terms of the microtubule cytoskeleton the authors would need to image microtubule invasions into spines (see here for example: https://pubmed.ncbi.nlm.nih.gov/27658622/).

We apologize for the confusion. TBA-1 is tagged on the N-terminus, but this was not labeled correctly in the figure. We have now fixed the figure labeling. The marker was generated from a construct provided by the Shen lab (Yan, J. et al. 2013, *eLife*). The distribution of the marker in DD neurons is similar to previously published findings for the cholinergic DA9 neuron.

Our main goal was to show GABAergic dendritic spines in worms are enriched with F-actin, in contrast to the main dendritic shaft which is enriched with microtubules. To be thorough, we also showed instances where GFP::TBA-1 signal is detectable at the base of individual spines (Figure S1B), but we did not intend to make specific claims about parallels to microtubule invasions observed for vertebrate spines. In the future it would be interesting to assess specific contributions of microtubules and microtubule related proteins to dendritic spine formation/maintenance, but we hope the reviewer will agree those experiments lie outside the scope of the current study. We have now modified the text to clarify these points.

2. The marker used to claim presence rough ER in SLPs seems highly overexpressed since it does not show ER pattern in the cell body. Similar to point (1), my view is that since the presence of ribosomes in SLPs was already shown by EM, this supplementary figure detracts rather adds to the paper. If the authors wish to make the claim about rough ER being in SLPs they should show more convincing data and explain why their data contrasts with other work that failed to detect rough ER in worm neurites (i.e Rolls&Rapoport).

We have now modified the figure to more clearly indicate TRAM-1 expression is concentrated in the cell body consistent with previous work (Rolls et al. MBoC, 2002). We also provide an additional image demonstrating more limited TRAM-1 localization in the dendrite, though we cannot completely rule out contributions from overexpression (Figure S1 F-H). We note that TRAM-1 localization to cholinergic motor neuron processes has been observed previously (for example, Edwards et al. *Genetics,* 2013). As the reviewer notes, ribosomes were previously identified near GABAergic spines by EM. Our work provides complementary evidence that rough ER markers may also localize in the synaptic region of GABAergic dendrites. We have modified the text to reflect these changes.

3. Can they show a more zoomed-out version of data in Figure 2A and B?

We have now included a zoomed-out version of panels from 2A and 2B in Figure S5.

4. Developmental timeline (wildtype versus neurexin): Is it possible to add a timepoint between 24hrs and L4 as well as one timepoint after L4? That would substantiate the claim of a stabilization function for Neurexin.

We have added new measurements of dendritic spines in control versus *nrx-1(wy778)* at 32 hours and in young adults in Figure 3 and Figure S9.

5. I did not understand the claim that "Receptor clustering followed a similar trend to spine formation" since in Figure 3E ACR-12::GFP seems affected already at 18hrs. Can they explain?

The reviewer is correct. The formation and disassembly of receptor clusters precedes spine formation/disassembly. We intended to highlight our observation that both receptor clusters and spines are formed in the absence of neurexin and then subsequently disappear. We have modified the text to clarify this point.

6. The analysis of how activity mutants affect spines (Table 1) is very nice. However, there is some discrepancy (for example unc-13 vs unc-18, where one is significant and the other isn't although presumably both regulate the same process). Can they add a column that note whether the alleles used nulls or weak/strong hypomorphs?

We have added a column in Table 1 describing the nature of each allele. We now also include new data showing that spine density is not significantly altered in L4 stage *unc-13(s69)* and *unc-17(e245)* mutants (see response to Reviewer 2).

7. The claims about UNC-104/KIF1A transporting neurexin should be modified to state that Neurexin localizes in an unc-104 dependent manner. To claim transport by unc-104 the authors should show more direct evidence such as co-transport with a known unc-104 cargo.

We agree with the reviewer and have modified the text accordingly.

Reviewer #2: This study by Oliver and colleagues explores the time course of postsynaptic spines at the GABAergic motor neurons in the nematode C. elegans. The findings provide significant insight into an important topic: how spine formation and maturation are determined. By analyzing the presynaptic organization and postsynaptic spine development, the authors presented trajectory of spine formation and maturation. The paper also indicates that cell adhesion molecule nrx-1/neurexin is a key component during spine maturation but is not required for spine formation. Moreover, unc-104/kinesin motor protein also plays important roles by transporting nrx-1 to proper sites. Overall, the study is methodical, experimentally sound, and the manuscript is well-written and logical. The manuscript presents some interesting findings but will however be strengthened considerably by addressing several key points as detailed below.
We thank the reviewer for their positive comments.

1. Is the spine structure GABAergic synapses specific in worm? The authors may need to give some background somewhere.

Though not yet characterized by fluorescent microcopy, John White and colleagues identified a limited number of other neuron classes that may have similar spine structures (J.G. White et al. 1976) (J.G. White et al., 1986) (J. White, 2018). We now include discussion of these findings.

2. Page9. Ca2+ responses in spines were reduced by 80% in unc-17 mutants. What are the 20% remaining? Is this evoked by other neurotransmitters from cholinergic neurons? This question was asked because cholinergic synaptic transmission is completely abolished in the NMJs of the unc-17 mutants.

Though we are not certain, we speculate that this remaining signal may arise from release of other neurotransmitter or neuropeptide signals onto GABAergic neurons. It is also possible that these responses reflect evoked ACh release from motor neuron classes where *unc-17* expression is less strongly affected by the *e113* mutation.

3. Since spines on the GABAergic motor neurons have different shapes (Figure 1B), how is the spine length measured in Table 1?

Spine length is measured from the base of the spine to the tip of the longest part of the protrusion. For example, in branched spines, only one measurement was taken from the base of the spine to the tip of the longest branch.

4. Page 13. The authors should be careful when saying that the postsynaptic spine formation is not highly related to the presynaptic activity. The mutants that block presynaptic SV exocytosis, such as unc-13 and unc-18, were used. Based on the results from NMJs, a substantial spontaneous release can still occur in unc-13 (e51) and unc-18 mutants. The low frequency

spontaneous events may be sufficient to trigger spine formation. The author may need to consider using the unc-13(s69) mutant which is almost null and no spontaneous release occurs (Hu et al, 2013, eLife). Unc-31 mutant is not a good example as the spontaneous release is unaltered (Gracheva et al., 2007, JNS). The observation in unc-17 mutants is somewhat surprising. Is it possible that other neurotransmitters are also involved? The spine number and length are both significantly reduced in acr-2 gf mutants, but the authors didn't give an description for the results. In Zhou's paper (2017, Cell Reports), spontaneous release occurs normally in this mutant. Overall, it will be interesting to know how spine formation is affected in mutants that completely block transmission, or on the contrary, that have increased presynaptic activity.

We thank the reviewer for this comment. We included *unc-31* in our analysis to explore potential involvement of DCV release and make no claims about synaptic vesicle release in this mutant. However, we agree that additional analysis was warranted and, as suggested by the reviewer, now include new data showing that spine density in L4 stage *unc-13(s69)* animals is similar to wild type. Our combined studies of *unc-17*, *unc-13* and *unc-18* mutants are most consistent with the idea that synaptic activity is not absolutely required for spine formation, though future studies using cell-specific approaches for blocking neurotransmission could potentially refine this understanding further.

Dendritic spines were unaffected in *acr-2(ok1887)* null animals, but were reduced in *acr-2(gf)* animals. At this point we don't have a clear understanding of the cause of this reduction. Prior transcriptome analysis of cholinergic motor neurons in *acr-2(gf)* animals has indicated significant gene expression changes as a consequence of this mutation, in particular neuropeptide related genes (McCulloch et al, 2020). An interesting possibility is that gene expression changes in cholinergic motor neurons of *acr-2(gf)* animals affect signaling pathways important for spine outgrowth/stabilization. We now include discussion of this point.

5. In Figure3A, the number of the NRX-1::GFP puncta is almost the same with the spine number, while the spine number is significantly lower than the presynaptic puncta of CLA::GFP or SNB-1::GFP. The author may need to explain the differences.

We thank the reviewer for pointing this out. The difference in CLA-1/SNB-1 and NRX-1 puncta relative to the number of spines may suggest that some presynaptic release sites lack NRX-1 and are not associated with clear dendritic spines; however, we note that that some of these presynaptic puncta may represent trafficking vesicles rather than mature release sites. Likewise, the variable size/shape of spines makes them more difficult to quantify than presynaptic puncta. We have modified the text to include discussion of these points.

6. Is the nrx-1 wy778 a null mutant? If worm also expresses different isoforms of nrx-1, are they all deleted in wy778 allele?

The *nrx-1* gene encodes long α and short γ isoforms. The *wy778* allele we used in our study deletes the C terminal and transmembrane regions that are common to both isoforms and is believed to be a null allele. To further address this issue, we have included new data showing

that dendritic spines are also absent in L4 stage *nrx-1(wy1155)* null animals (Figure S7) where the entire *nrx-1* open reading frame is deleted (Kurshan, P., et al., *Neuron,* 2018).

7. Although the authors presented clear evidence that nrx-1 is involved in spine maturation, they didn't mention the important binding partner of nrx-1, nlg-1/neuroligin. Is nlg-1 localized in the postsynaptic GABAergic spines? It will be interesting to know whether the observed phenotypes in nrx-1 mutants are also nlg-1 dependent.

Our prior work showed that deletion of *nlg-1* does not significantly alter either dendritic spines or receptor clusters in GABAergic neurons (Philbrook et al., 2018). Notably, prior studies of *nlg-1* reporters (Staab et al., 2014; Hunter et al., 2010) and more recent transcriptome profiling efforts (Taylor et al. 2021) show that *nlg-1* is not strongly expressed in GABAergic motor neurons. We are continuing to investigate potential interactions with alternate NRX-1 binding partners. We include expanded discussion of these points in the revised version of the text.

8. How does unc-104/kinesin transport nrx-1 to presynaptic sites? Do they bind to each other? Do both the long and short isoforms of nrx-1 have the transmembrane domain? Are both isoforms required for spine maturation?

A physical interaction between neurexin and KIF1A has not yet been defined. There are several models for how UNC-104 may mediate trafficking. For example, the LBD (liprin-α binding domain) motor domain of KIF1A can interact with liprin-α which associates with cargo vesicles (Shin, H., et al. 2003). In addition, the C-terminal pleckstrin homology (PH) domain interacts with the phosphatidylinositol(4,5)bisphosphate lipids on cargo vesicles (Klopfenstein, D.R., et al. *Cell,* 2002). The *unc-104(e1265)* allele used in our study carries a point mutation in the PI(4,5)P2 binding pocket of the PH domain and reduces the ability of UNC-104 to bind to cargo (Kumar et al, 2010). Our analysis therefore implicates this domain in UNC-104-dependent localization of NRX-1.

Both long and short NRX-1 isoforms include the transmembrane region. Our prior investigation of *nrx-1(nu485)* mutants implicate the long isoform is required for synapses onto GABAergic neurons (Philbrook et al. 2018). *nrx-1(nu485)* mutants carry a deletion that affects the extracellular domain of the long isoform (leaving the short isoform intact). Further dissection of their respective functions will require mutant alleles that selectively impact expression of the short isoform.

Reviewer #3: PGENETICS-D-21-01188 "Kinesin-3 mediated axonal delivery of presynaptic neurexin stabilizes dendritic spines and postsynaptic components"
Dendritic spines, the small protrusions from the dendrite membrane, are the contact site with neighboring axons where synaptic input is received. Recent advances in microscopy have uncovered that also in C. elegans, spine like protrusions of GABAergic motor neurons exhibit some of the hallmarks of mammalian dendritic spines. In this study, Oliver et al focus on the developmental aspects of the formation of post synaptic specialization sites of GABAergic motor neurons. The authors nicely define the order and timing of the formation of the pre and post synaptic sites, and demonstrate that NRX-1 is required for stabilization, but not initial formation of the dendritic spines. The kinesin UNC-104 transports NRX-1 and its activity is required for

synapse maturation and stabilization. The last experiments shown in figure 5 of temperature shifts of the unc-104 mutants nicely teases out the temporal aspects of the requirement of UNC-104 delivery of NRX-1 for postsynaptic maturation and maintenance of mature spines. Overall this is an interesting manuscript with important and thorough findings, that are significant for our understanding of the functional properties of neural circuits. My specific comments can be found below.

We thank the reviewer for their positive assessment.

My main point goes toward emphasizing the novelty of this paper beyond the previous two recently published studies. Philbrook et al 2018 and Cuentas-Condori et al 2019 both describe the functional aspects of the GABAergic dendritic spines in C. eleagns. Cuentas-Condori defines these structures, the cytoskeletal properties and the calcium transients triggered by presynaptic activity. Philbrook at, besides describing these structures, also demonstrates that NRX-1, located at presynaptic sites, specifically directs postsynaptic development. The authors do go beyond these two manuscripts, but in my opinion often fail to distinguish previous already published findings from new unpublished data. To give one example, Figure 1 is highly redundant with previous studies and I almost do not see anything that wasn't already described. Why do the authors characterize spine morphology in Figure 1B? it was already characterized in Cuentas-Condori et al and the authors don't use it again later on in the manuscript (they could, for example in table 1 of receptor analysis). This point could be addressed by some rewriting to highlight novel points and shorten where redundancy occurs.

We thank the reviewer for this suggestion. We have rearranged Figure 1 to emphasize new findings and moved panel B to supplement. We have also shortened the text as suggested and make every effort to distinguish new findings from confirmatory findings. We note that the papers cited by the reviewer are the only 2 prior publications characterizing GABAergic spines in worms. Though some elements of Figure 1 and supplements might be considered confirmatory, we suggest that they remain quite important for the field given the relative newness of this area of study.

Minor points:
1. In Figure 1 panel I (calcium imaging), please explain why the Gaussian fitting was used.

We measured GCaMP fluorescence over a 5 s period of continuous Chrimson stimulation. Based on analysis of residuals, we found that a Gaussian fit reliably profiled the calcium response during this window and provided more accurate quantification of the peak compared with other approaches for peak detection that involve averaging or line smoothing. We used a similar analysis approach in previous work (Philbrook et al, 2018).

2. In Figure 4, please mention the developmental stage of the imaged worms (I assume its L4).

The developmental stages are now indicated in the figure caption.

3. In Figure 4, the title of the figure could be rephrased: the evidence presented in this figure support the requirement of nrx-1 to synapse formation or stabilization but not necessarily stabilization.

We have modified the title accordingly.

4. In Figure S4.5, what the authors call 'modest decrease in axonal NRX::GFP' in unc-116 mutants, is actually a reduction in a third of the signal. I suggest that the authors at least raise possible explanation as to why the residual activity of UNC-116 does not appear in unc-104 mutants.

We thank the reviewer for pointing this out. Our analysis indicates NRX-1 localization to synapses is strongly UNC-104-dependent. However, mutation of UNC-116 also produces a less severe reduction in NRX-1 localization to synapses. The synaptic localization of several pre- and postsynaptic proteins have previously been shown to have a partial dependence on UNC-116 (Hoerndli et al. 2013; Byrd et al. 2001). We therefore speculate that UNC-116 may indirectly contribute to NRX-1 synaptic localization through effects on other synapse-associated proteins. We have modified the text to include discussion of this point.

5. In Figure 5, it would add extra value and novelty to the Figure if the authors prolonged the experiment and added one additional shift back to the permissive temperature after L4, to address whether de novo formation of NRX-1 synapses is possible during adulthood.

This is an interesting suggestion and we have now performed the experiment. We found that there was not a significant difference in spine density for *unc-104(ts)* animals grown to adulthood continuously at 13.5°C versus animals that were transiently switched to the restrictive temperature during adulthood and then returned to the permissive temperature. These results suggest there is some recovery of spines during adulthood. However, we note that this was a challenging experiment to perform and we are therefore very cautious in our interpretation. Imaging dendritic spines in adults is difficult compared to L4 animals (in part due to intestinal autofluorescence). In addition, we noted a severe reduction in spine density even in control *unc-104(ts)* animals grown at the permissive temperature, complicating interpretation. We include a figure describing these findings below for reviewers but at present do not include it in the revised submission.

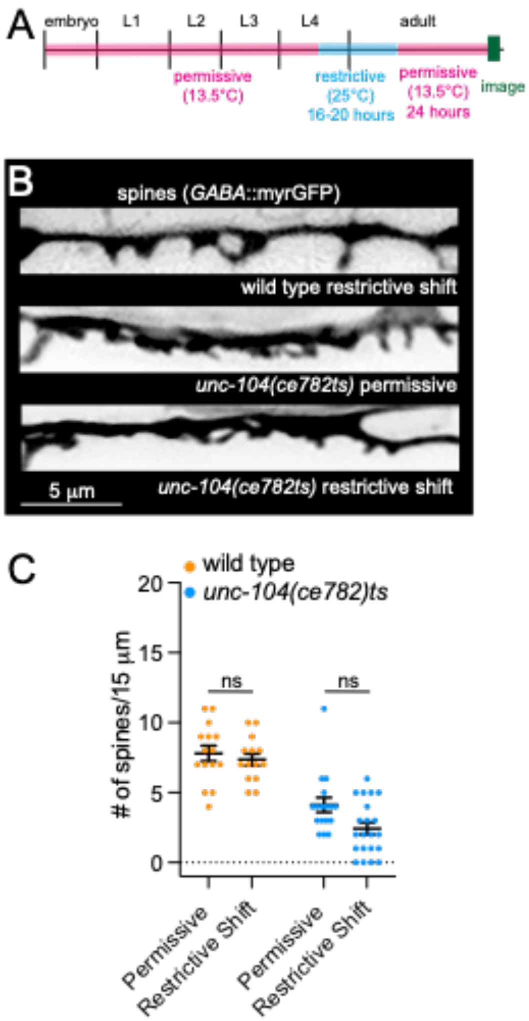

Animals were grown at 13.5°C until L4 stage before a shift to the restrictive temperature of 25°C for 16-20 hours, then returned to the permissive temperature for 24 hours before imaging. We found that there was not a significant difference in spine density for *unc-104(ts)* animals grown to adulthood continuously at 13.5°C versus animals that were transiently switched to the restrictive temperature during adulthood and then returned to the permissive temperature. Taken together with results reported in Figure 5E, these findings suggest there may be some recovery of spines with shift to permissive temperature during adulthood.

6. The supplemental figure list is extremely long. The authors can easily combine many of the supplementary figures into denser versions, for a better presentation of the figures and a smoother reading.

We made every effort to condense the supplemental figures while also incorporating additional data requested by reviewers. There are now a total of 14 figure supplements in the revised version.
