# Supplementary material for: Kinesin-3 mediated axonal delivery of presynaptic neurexin stabilizes dendritic spines and postsynaptic components
Source: PLoS Genet. 2022 Jan 28;18(1):e1010016. doi: 10.1371/journal.pgen.1010016 (PMC8827443; doi:10.1371/journal.pgen.1010016)
Supplement: Attachment — Submitted filename: Paper review.docx [file pgen.1010016.s023.docx]

PGENETICS-D-21-01188 “Kinesin-3 mediated axonal delivery of presynaptic neurexin stabilizes dendritic spines and postsynaptic components”

Dendritic spines, the small protrusions from the dendrite membrane, are the contact site with neighboring axons where synaptic input is received. Recent advances in microscopy have uncovered that also in *C. elegans*, spine like protrusions of GABAergic motor neurons exhibit some of the hallmarks of mammalian dendritic spines. In this study, Oliver et al focus on the developmental aspects of the formation of post synaptic specialization sites of GABAergic motor neurons. The authors nicely define the order and timing of the formation of the pre and post synaptic sites, and demonstrate that NRX-1 is required for stabilization, but not initial formation of the dendritic spines. The kinesin UNC-104 transports NRX-1 and its activity is required for synapse maturation and stabilization. The last experiments shown in figure 5 of temperature shift of the *unc-104* mutants nicely teases out the temporal aspects of the requirement of UNC-104 delivery of NRX-1 for postsynaptic maturation and maintenance of mature spines. Overall this is an interesting manuscript with important and thorough findings, that are significant for our understanding of the functional properties of neural circuits. My specific comments can be found below.

My main point goes toward emphasizing the novelty of this paper beyond the previous two recently published studies. Philbrook et al 2018 and Cuentas-Condori et al 2019 both describe the functional aspects of the GABAergic dendritic spines in *C. eleagns.* Cuentas-Condori defines these structures, the cytoskeletal properties and the calcium transients triggered by presynaptic activity. Philbrook at, besides describing these structures, also demonstrates that NRX-1, located at presynaptic sites, specifically directs postsynaptic development. The authors do go beyond these two manuscripts, but in my opinion often fail to distinguish previous already published findings from new unpublished data. To give one example, Figure 1 is highly redundant with previous studies and I almost do not see anything that wasn’t already described. Why do the authors characterize spine morphology in Figure 1B? it was already characterized in Cuentas-Condori et al and the authors don’t use it again later on in the manuscript (they could, for example in table 1 of receptor analysis). This point could be addressed by some rewriting to highlight novel points and shorten where redundancy occurs.

Minor points:

1. In Figure 1 panel I (calcium imaging), please explain why the Gaussian fitting was used.
2. In Figure 4, please mention the developmental stage of the imaged worms (I assume its L4).
3. In Figure 4, the title of the figure could be rephrased: the evidence presented in this figure support the requirement of nrx-1 to synapse formation or stabilization but not necessarily stabilization.
4. In Figure S4.5, what the authors call ‘modest decrease in axonal NRX::GFP’ in *unc-116* mutants, is actually a reduction in a third of the signal. I suggest that the authors at least raise possible explanation as to why the residual activity of UNC-116 does not appear in *unc-104* mutants.
5. In Figure 5, it would add extra value and novelty to the Figure if the authors prolonged the experiment and added one additional shift back to the permissive temperature after L4, to address whether *de novo* formation of NRX-1 synapses is possible during adulthood.
6. The supplemental figure list is extremely long. The authors can easily combine many of the supplementary figures into denser versions, for a better presentation of the figures and a smoother reading.
